# Perioperative anaphylaxis due to intravenous tranexamic acid following cutaneous exposure

**DOI:** 10.1186/s40981-025-00840-6

**Published:** 2025-12-10

**Authors:** Yasuhiro Amano, Takahiro Tamura, Yuko Konishi, Yui Somura, Koichi Akiyama

**Affiliations:** 1 Department of Anesthesiology, Nagoya University Graduate School of Medicine, 65 Tsurumai-cho, Showa-ku, 466-8550 Nagoya, Japan; 2 Endowed Division of Perioperative Management, Nagoya University Graduate School of Medicine, Nagoya, Japan

**Keywords:** Tranexamic acid, Perioperative anaphylaxis, Cutaneous sensitization

## Abstract

**Background:**

Tranexamic acid (TXA) is widely used to prevent surgical bleeding; further, it has been recently applied in cosmetic dermatology. TXA-induced anaphylaxis is very rare; however, most reported cases occur upon initial administration.

**Case presentation:**

This case report describes a 41-year-old man with atopic dermatitis who developed TXA-induced anaphylaxis during induction of general anesthesia for spinal surgery. Intradermal testing using TXA yielded reproducible positive reactions, while other tested drugs yielded negative results. His medical history revealed repeated episodes of dermatitis 1 year prior after applying a topical skin-lightening cream containing TXA. This suggested that prior percutaneous exposure may have contributed to sensitization.

**Conclusions:**

Our findings suggest a possible relationship between prior topical exposure to TXA and perioperative anaphylaxis. Although TXA-induced anaphylaxis is extremely rare, the increasing use of TXA in both surgical and cosmetic contexts may increase opportunities for exposure. Therefore, clinicians should be aware of this potential risk.

## Background

Tranexamic acid (TXA) is a synthetic lysine analogue widely used to reduce surgical bleeding in various operative settings. It exerts antifibrinolytic effects by inhibiting the conversion of plasminogen to plasmin, thereby suppressing fibrin degradation. Recently, its application has expanded beyond perioperative hemostasis to include cosmetic dermatology, especially in the treatment of melasma and other pigmentary disorders. Despite its increasing use in this field, the mechanisms by which TXA improves hyperpigmentation remain incompletely understood. Current evidence suggests that TXA modulates plasmin-mediated inflammatory signaling and reduces the expression of melanogenic mediators, including endothelin-1 and pro-inflammatory cytokines, and suppresses melanocyte activity [1]. Despite the broad application and overall favorable safety profile of TXA, TXA-induced anaphylaxis may occur in extremely rare cases. Large-scale epidemiological studies have described rare cases of TXA-induced perioperative anaphylaxis [2–6]; further, a recent systematic review identified only about 15 reports of such cases, which mostly occurred upon the initial intravenous administration [7]. Given its rarity, TXA-induced anaphylaxis may not be initially suspected in perioperative settings; however, a positive allergy test result should prompt careful reconsideration of TXA as a potential culprit rather than being dismissed solely based on low prior probability.

The present article describes a case where a patient developed anaphylaxis shortly after intravenous TXA administration during anesthesia induction for spinal surgery. Although cefazolin was initially suspected, detailed evaluation including skin testing ultimately implicated TXA. Notably, the patient had a history of repeated dermatitis following the application of an over-the-counter skin-lightening cream that contained TXA, which suggested the possibility of percutaneous sensitization. This case highlights a potential but underrecognized pathway of TXA sensitization that could contribute to perioperative anaphylaxis.

## Case presentation

**Fig. 1:**
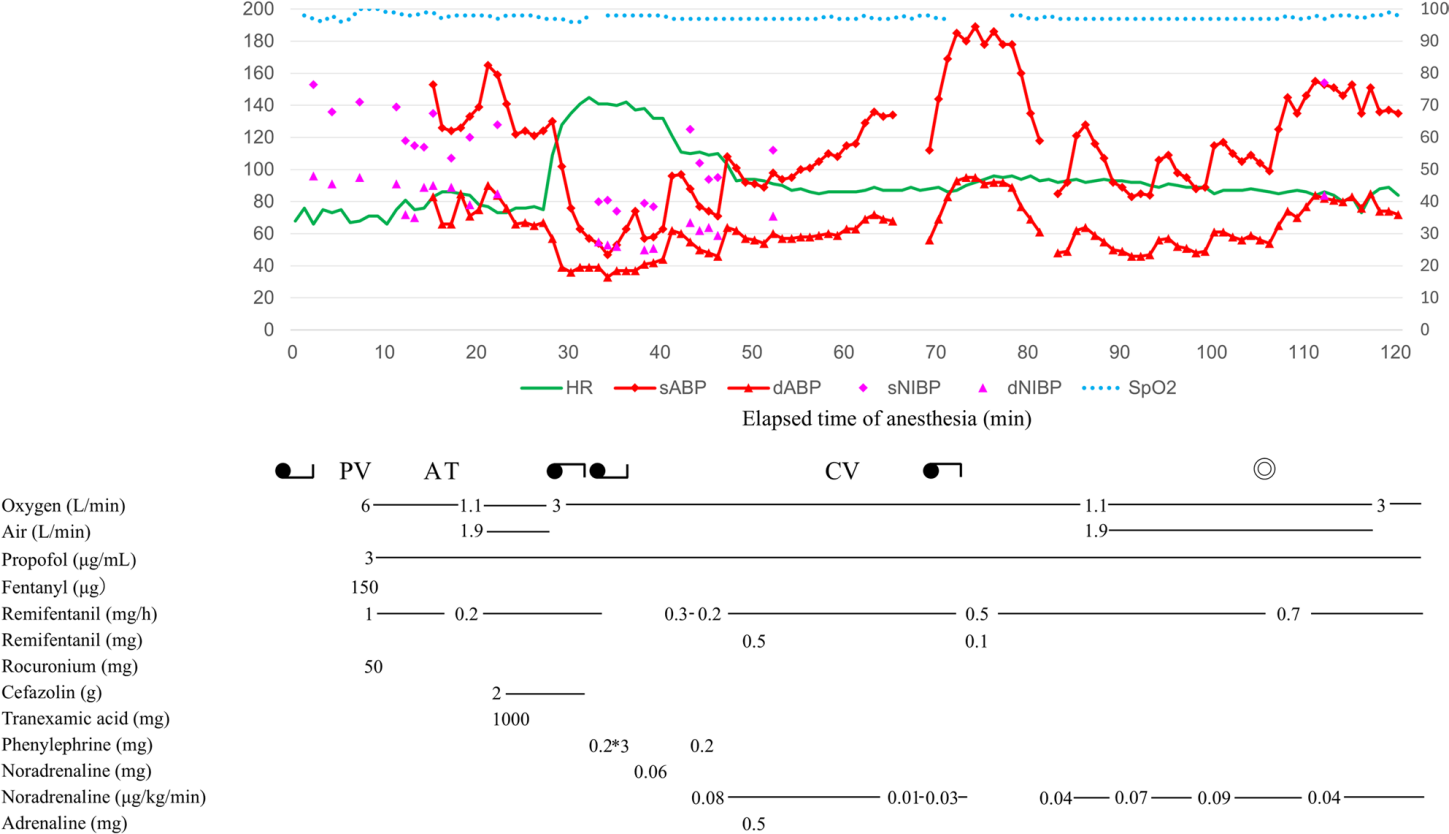
Anesthetic chart from general anesthesia induction to the onset of anaphylaxis and subsequent hemodynamic stabilization HR, heart rate; sABP, systolic aortic blood pressure; dABP, diastolic aortic blood pressure; sNIBP, systolic non-invasive blood pressure; dNIBP, diastolic non-invasive blood pressure; PV, peripheral catheter insertion; A, arterial catheter insertion; T, intubation; CV, central venous catheter insertion; ◎, start of surgery

A 41-year-old man, measuring 172 cm in height and weighing 75 kg, was scheduled to undergo resection of an intradural extramedullary spinal tumor at the thoracolumbar junction. He had atopic dermatitis and was receiving topical corticosteroid therapy. He also had a history of walnut allergy. General anesthesia was induced using propofol, fentanyl, remifentanil, and rocuronium. Tracheal intubation was performed uneventfully. Subsequently, after disinfection with povidone-iodine, a latex-free urinary catheter was inserted. Cefazolin administration was initiated. After 3 min, TXA was administered, and the patient was placed in the prone position. At 5 min after initiation of cefazolin administration, the patient developed hypotension and tachycardia (Fig. 1). Within 10 min, the systolic blood pressure had decreased to 54 mmHg, with cefazolin administration having been completed by this point. Despite repeated phenylephrine administration, hypotension persisted. Next, the patient was returned to the supine position. A bolus of 60 µg noradrenaline was administered, followed by initiation of continuous infusion. Immediately thereafter, generalized flushing was observed. Due to suspected anaphylaxis, 0.5 mg intramuscular adrenaline was administered. A central venous catheter was inserted; after confirmation of hemodynamic stability following repositioning to the prone position, surgery was initiated. Cefazolin was suspected to be the cause of anaphylaxis, and perioperative antibiotics were withheld. The surgery proceeded as planned, and noradrenaline infusion was discontinued. The patient was extubated in the operating room and transferred to the surgical intensive care unit for observation. The next day, the patient was returned to the ward; he was discharged on postoperative day 10. Plasma histamine and tryptase levels were substantially elevated at 30 min after symptom onset (2.97 µg/L and 14.5 µg/L, respectively) compared with those after 24 h (0.38 µg/L and 3.6 µg/L) [8, 9].

**Fig. 2:**
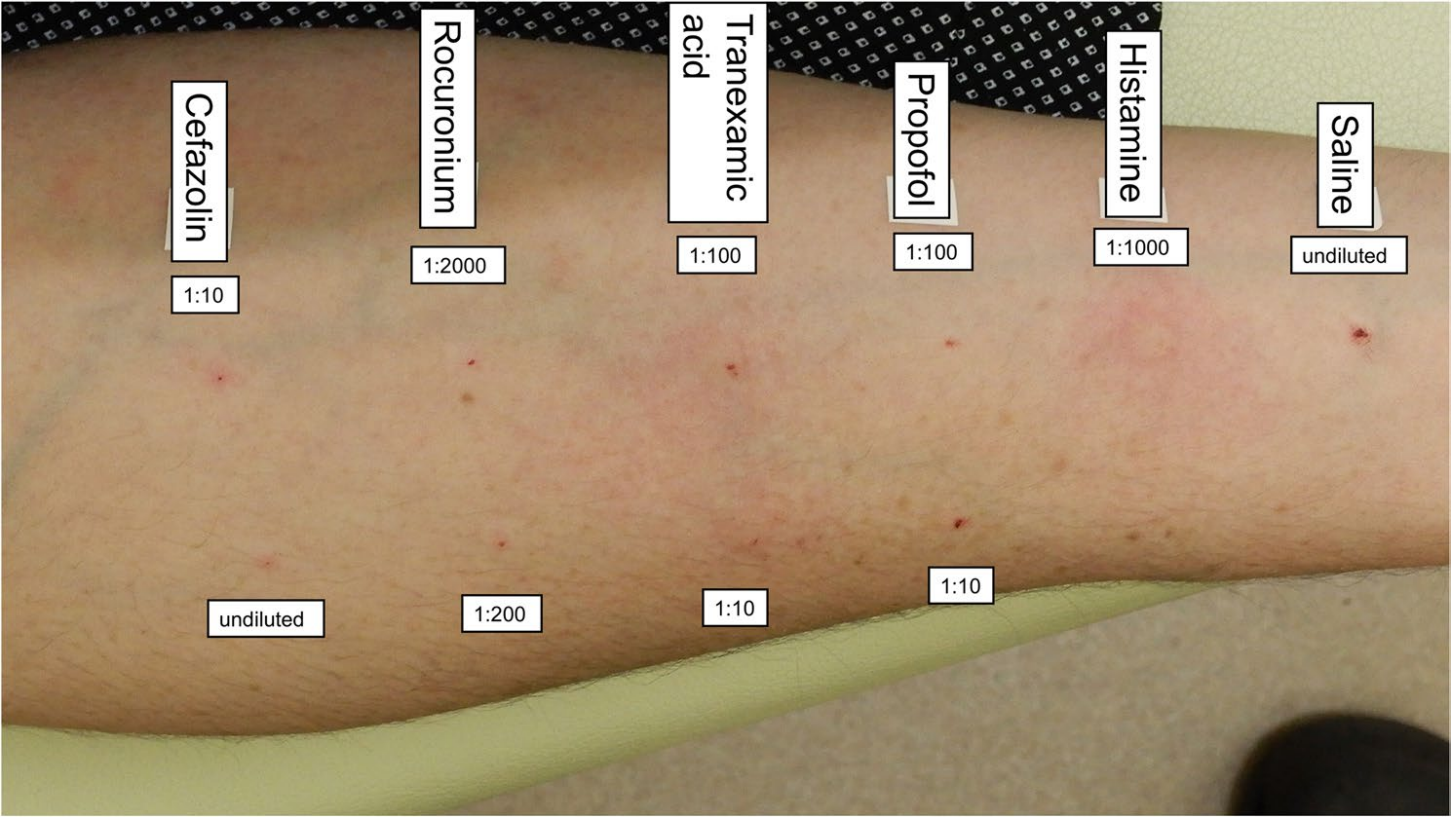
Intradermal test results (forearm). The names and concentrations for each drug used in the intradermal tests are shown

**Fig. 3:**
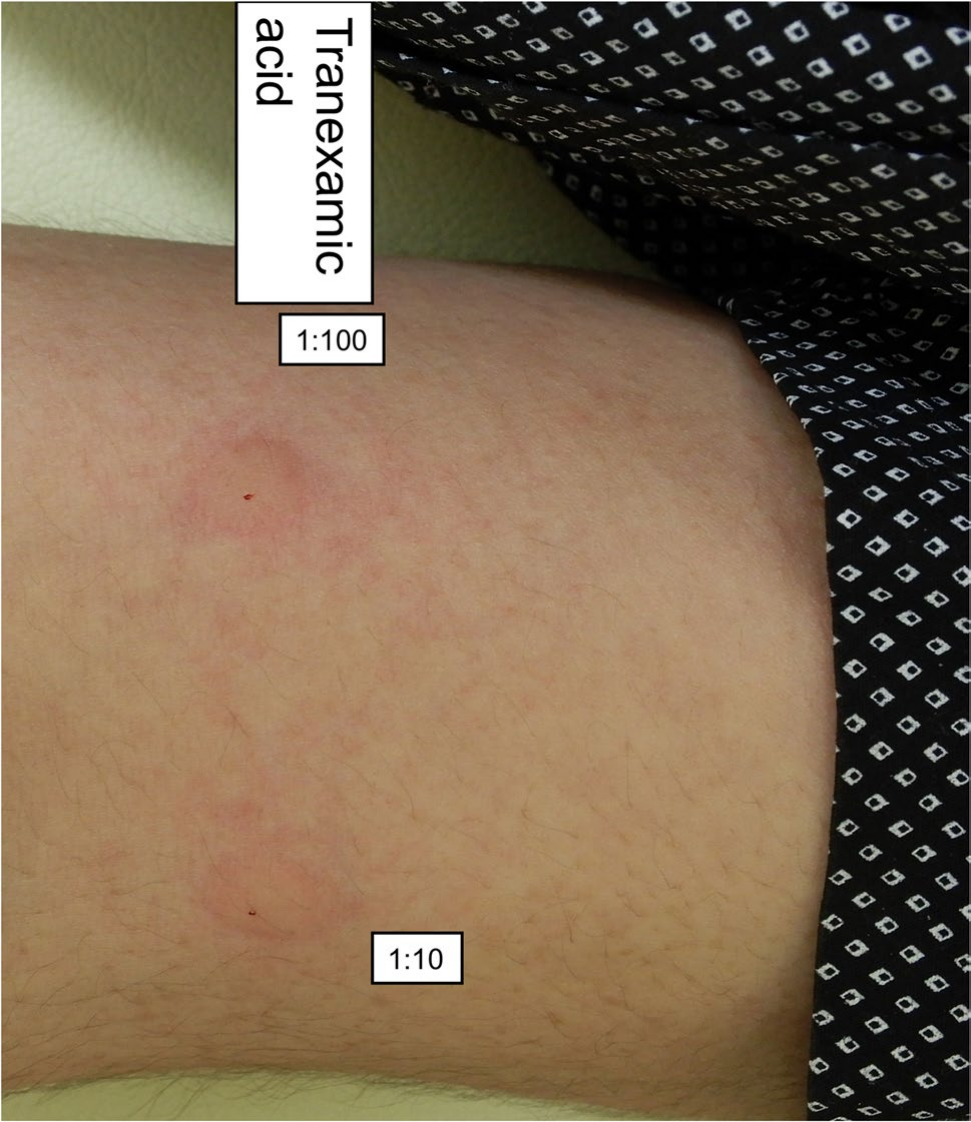
Additional intradermal test results for tranexamic acid (upper arm)

After 1 month, skin testing was performed using propofol, TXA, rocuronium, cefazolin, and povidone-iodine. All drugs were negative on skin prick testing. In the intradermal test (IDT), cefazolin was negative, while TXA was positive at two concentrations (Fig. 2). A repeat IDT with a new vial of TXA similarly yielded positive reactions at these concentrations (Table 1; Fig. 3). No basophil activation was noted on testing, even with anti-IgE stimulation, which indicated a non-responder phenotype [10]. Therefore, TXA-induced anaphylaxis was confirmed.

**Table 1:** Intradermal test results

Drug	Dilution	Wi (mm)	W20 (mm)	Flare (mm)	Judgement
Saline 9 mg/mL	Undiluted	4.5	4.0	0	-
Histamine 10 mg/mL	1:1000	3.6	7.8	23.0	+
Propofol 10 mg/mL	1:100	4.1	4.5	0	-
	1:10	3.5	4.1	0	-
Tranexamic acid 100 mg/mL (1st)	1:100	3.3	6.6	25.0	+
	1:10	3.3	7.4	20.6	+
Rocuronium 10 mg/mL	1:2000	4.2	4.7	0	-
	1:200	5.1	3.0	0	-
Cefazolin 10 mg/mL	1:10	3.5	3.1	0	-
	Undiluted	3.9	2.5	0	-
Tranexamic acid 100 mg/mL (2nd)	1:100	4.4	10.0	24.8	+
	1:10	4.1	9.4	24.7	+

Wi, diameter of the initial wheal just after injection; W20, diameter of the wheal 20 min post-injection

Saline and histamine were used as the negative and positive controls, respectively. Povidone iodine was excluded in the intradermal test [11]. Saline was used for dilution, and the concentrations of each drug were set according to two guidelines at the recommended maximum concentration and a tenfold diluted concentration [11, 12]. The injection volume for the intradermal test was 0.02 mL for all drugs and a 3–5 mm wheal was made on the patient’s forearm. The diameters of the wheals and flares were measured using digital calipers. A positive reaction was judged based on the following criteria [12]: W20 ≥ Wi + 3 mm with surrounding flare. Intradermal tests with tranexamic acid at 1 mg/mL and 10 mg/mL yielded positive results. To exclude the possibility of a preparation error, a second intradermal test with a new vial of tranexamic acid was performed on the patient’s upper arm, which again yielded positive reactions at the same concentrations

Given the extreme rarity of TXA-induced anaphylaxis [7], we obtained additional history. The patient reported having repeatedly used an over-the-counter skin-lightening cream approximately one year before surgery, which resulted in severe facial dermatitis each time. The cream was found to contain TXA. This suggested the possibility of cutaneous sensitization through prior topical exposure, which might have contributed to the subsequent anaphylactic reaction following intravenous administration. The patient was instructed to strictly avoid TXA in all forms, including oral, intravenous, and topical preparations.

## Discussion

The causative agent of perioperative anaphylaxis should not be solely determined by the temporal association with drug administration [13]. Nonetheless, tentative causative agents can be proposed before establishing the definitive agent when feasible, while avoiding unnecessary changes to all other drugs. In the present case, neurophysiological monitoring required propofol and opioids [14], which have few suitable substitutes; however, remimazolam has been shown to allow stable monitoring and may be an alternative to propofol [15, 16]. Anaphylaxis occurred 20 min after administration of propofol, fentanyl, remifentanil, and rocuronium; 5 min after cefazolin; and 2 min after TXA. Since intravenous drugs most often trigger anaphylaxis within 15 min [3, 17], cefazolin and TXA were relatively more plausible causative agents. Since cefazolin is among the most common causes of perioperative anaphylaxis in Japan [2, 18], it was initially suspected. However, we established TXA as the definitive causative agent.

Generally, TXA is considered a safe drug and is included in the World Health Organization’s list of essential medicines [19]. Adverse reactions to TXA are uncommon and are typically mild to moderate, including thrombosis or seizures [20]. TXA-induced anaphylaxis is extremely rare, with a recent systematic review identifying 15 reported cases worldwide [7]. Accordingly, the possibility of false-positive skin test results must always be considered. In the present case, skin testing was performed based on two guidelines [11, 12], which recommend maximum concentrations to minimize false positives. For TXA, the suggested maximum concentration for IDT is 10 mg/mL. In our patient, we performed IDT using TXA concentrations at 1 mg/mL and 10 mg/mL, which both yielded positive reactions on two separate occasions. However, IDT with 10 mg/mL of TXA has been shown to occasionally yield false positives in healthy volunteers; contrastingly, all participants yielded negative results with 2 mg/mL [21]. Since the recommended maximum concentration for IDT should be the highest concentration that does not induce false positives in healthy individuals [22], our finding with 10 mg/mL may have been a false positive. Nonetheless, reproducible positive reactions at 1 mg/mL, which fall below the 2 mg/mL threshold, confirmed TXA as the causative agent. Current Japanese guidelines for perioperative anaphylaxis provide important recommendations [23]; however, they do not cover all concentrations for skin testing of individual drugs. Accordingly, further refinement is warranted. The basophil activation test is a flow cytometry-based in vitro assay that assesses basophil degranulation in response to specific allergens; in this case, basophil activation test was performed using CD203c as the activation marker. Our patient showed no increase in CD203c expression even with anti-IgE stimulation, indicating a non-responder phenotype rather than true absence of sensitization [10]. Given the positive IDT and occurrence of anaphylaxis at 2 min after TXA administration, the temporal relationship was consistent with the skin test findings, which confirmed TXA as the definitive causative agent.

Once TXA was identified as the causative agent, we considered the possibility of cross-reactivity with structurally related compounds. Intravenous TXA does not contain excipients; moreover, since it undergoes minimal metabolism, with >95% of it being excreted unchanged in urine [24], the antigen is most likely TXA itself. Although other lysine derivatives may cross-react, and thus should be avoided [25], they are not clinically used in Japan; therefore, only TXA needs to be avoided. As a synthetic lysine analog, TXA may cross-react with structurally related molecules, which may explain the occurrence of some cases of TXA-induced anaphylaxis at first known exposure [7]. In our patient, detailed history-taking revealed repeated use of a TXA-containing skin-lightening cream, which caused dermatitis. These findings suggest that cutaneous sensitization may have developed through prior topical exposure, with subsequent intravenous administration triggering anaphylaxis. A similar mechanism has been described with clindamycin, where topical exposure in patients with atopic skin was suspected to induce sensitization, which subsequently caused anaphylaxis upon intravenous administration [26]. To our knowledge, prior topical exposure to TXA leading to sensitization and subsequent anaphylaxis upon intravenous administration has not been previously reported.

The efficacy and safety of TXA is well established [24, 27–29]. Accordingly, it is being increasing used in dermatology and the expanding cosmetic-grade market [30, 31], which increases opportunities for cutaneous exposure, and thus raises the theoretical possibility of percutaneous sensitization. Further surveillance is warranted to provide epidemiological evidence for this pathway.

TXA-induced anaphylaxis remains an exceptionally rare but clinically significant event. Our case highlights that prior topical exposure may contribute towards sensitization even though we could not establish causality. With the increasing use of TXA in both surgical and cosmetic contexts, clinicians may need to remain vigilant and inquire about prior topical use when evaluating unexplained perioperative anaphylaxis.

## Data Availability

Data relevant to this case report are not publicly available because of concerns regarding patient privacy but are available from the corresponding author upon reasonable request.

## Abbreviations

IDT: Intradermal test
TXA: Tranexamic acid
IgE: Immunoglobulin E
